# Deep Brain Stimulation: Mechanisms, Cost-Effectiveness, and Precision Applications Across Neurology and Psychiatry

**DOI:** 10.3390/biomedicines13112691

**Published:** 2025-11-01

**Authors:** Horia Petre Costin, Felix-Mircea Brehar, Antonio-Daniel Corlatescu, Viorel Mihai Pruna

**Affiliations:** 1 Faculty of Medicine, “Carol Davila” University of Medicine and Pharmacy, 030167 Bucharest, Romania; horia-petre.costin0720@stud.umfcd.ro (H.P.C.); antonio.corlatescu0920@stud.umfcd.ro (A.-D.C.);; 2 Department of Neurosurgery, Clinical Emergency Hospital “Bagdasar-Arseni”, 041915 Bucharest, Romania

**Keywords:** deep brain stimulation, Parkinson’s disease, cost-effectiveness, subthalamic nucleus, globus pallidus internus, psychiatric disorders, complications, connectomics, artificial intelligence, neuromodulation mechanisms

## Abstract

In less than 30 years, Deep Brain Stimulation (DBS) has evolved from an antiparkinsonian rescue intervention into a flexible neuromodulatory therapy with the potential for personalized, adaptive, and enhancement-focused interventions. In this review we collected evidence from seven areas: (i) modern eligibility criteria, and ways to practically improve on these, outside of ‘Core Assessment Program of Surgical Interventional Therapies in Parkinson’s Disease’ (CAPSIT-PD); (ii) cost-effectiveness, where long-horizon models now show positive incremental net monetary benefit for Parkinson’s disease, and rechargeable-devices lead the way in treatment-resistant depression and obsessive–compulsive disorder; (iii) anatomical targets, from canonical subthalamic nucleus (STN) / globus pallidus internus (GPi) sites, to new dual-node and cortical targets; (iv) mechanistic theories from informational lesions, antidromic cortical drive, and state-dependent network modulation made possible by optogenetics and computational modeling; (v) psychiatric and metabolic indications, and early successes in subcallosal and nucleus-accumbens stimulation for depression, obsessive–compulsive disorder (OCD), anorexia nervosa, and schizophrenia; (vi) procedure- and hardware-related safety, summarized through five reviews, showing that the risks were around 4% for infection, 4–5% for revision surgery, 3% for lead malposition or fracture, and 2% for intracranial hemorrhage; and (vii) future directions in connectomics, closed-loop sensing, and explainable machine learning pipelines, which may change patient selection, programming, and long-term stewardship. Overall, the DBS is entering a “third wave” focused on a better understanding of neural circuits, the integration of AI-based adaptive technologies, and an emphasis on cost-effectiveness, in order to extend the benefits of DBS beyond the treatment of movement disorders, while remaining sustainable for healthcare systems.

## 1. Introduction

Deep Brain Stimulation (DBS) is a recognized and well-documented treatment for dopaminergic complications of Parkinson’s disease (PD) [1]. The most common DBS targets are the subthalamic nucleus (STN) and globus pallidus internus (GPi) [2,3]. The DBS introduction and its refinements help patients with pharmacologically uncontrollable motor fluctuations, levodopa-induced dyskinesias and drug-refractory PD tremors [4,5]. Therefore, DBS became an important therapeutic option for a selected category of treatment-resistant PD patients [6,7].

In the first five years after surgery, DBS may improve the motor features. There is also evidence that DBS can control the motor complications induced by levodopa for more than ten years [8]. Studies of STN-DBS versus GPi-DBS revealed improvements in motor features, including fluctuations, dyskinesia, on- and off-medication motor function, and activities of daily living at 36 months for both stimulation targets. STN-DBS also showed sustained reduction in levodopa equivalence daily dose (LEDD) [9].

**Figure 1:**
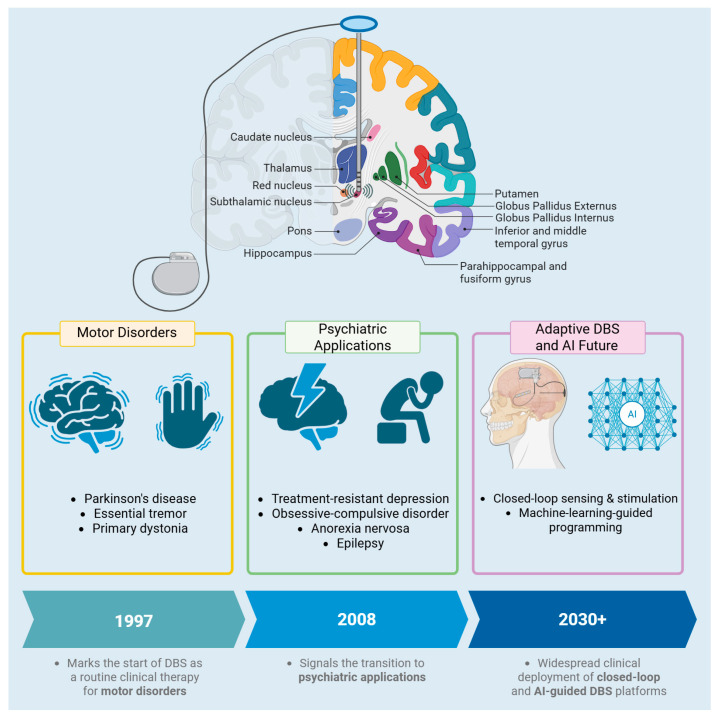
Evolution of DBS indications and technology, beginning as a therapy for PD and developing as a neuro-enhancement precision treatment for a variety of diseases, making use of still-emerging technologies. Created in BioRender https://BioRender.com/au28n2e (accessed on 8 September 2025).

DBS has been clinically utilized for almost 30 years. Yet, the selection criteria for the application of DBS in PD continue to be primarily rooted in the ‘Core Assessment Program of Surgical Interventional Therapies in Parkinson’s Disease’ (CAPSIT-PD), which was published in 1991 (Table 1) [10]. Unfortunately, the criteria have become more restrictive, given the vast advancements in the knowledge concerning PD, including understanding the PD course, phenotypic variability, and genotypic considerations, over nearly the last 30 years. CAPSIT-PD estimated that only 1.6% of persons with PD would be eligible for DBS, but it was estimated with more flexible and more inclusive criteria to just 4.5% [11] (Figure 1).

However, it is worth mentioning that, although only a small percentage of PD patients would be eligible for DBS surgery, studies have shown that DBS represents the most economically attractive option across long-horizon models, not only for the PD patients, but also for the patients who are in the psychiatric sphere.

A meta-analysis conducted by Lannon et al. (2024) [12] showed that DBS presents a positive incremental net benefit of USD 40,504.81 across studies with horizons of 15 years or longer, compared to the best medical therapy represented by the non-invasive pharmacotherapy, therefore proving the net beneficial cost-effectiveness of the DBS.

The results from a study by Nyholm et al. (2025) [13], registry-based modeling from Sweden, corroborated the information presented in the meta-analysis by tracking the costs of PD treatment over more than two decades of real-world data. The results were in favor of the DBS, which delivered more QALYs and with reduced total costs compared to the best medical therapy (represented by the levodopa treatment), continuous subcutaneous apomorphine injection and levodopa–carbidopa intestinal gel, when the researchers included in the model the caregivers and nursing home costs.

When taking into account just the direct medical costs, a study by Kabotyanski et al. (2024) [14] displayed an incremental cost-effectiveness ratio (ICER) of the DBS compared to the usual treatment for treatment-resistant depression of 31,878.61 USD/QALY. Taking into account the societal burden, which consists of caregivers and nursing homes, the ICER falls to −43,924.23 USD/QALY, meaning it is both cost-saving and more effective.

However, when it comes to the treatment of treatment-resistant obsessive–compulsive disorder, the type of DBS device has a decisive role. Najera et al. (2025) [15] found that the 5-year ICER for non-rechargeable DBS is 203,202 USD/QALY, while for rechargeable DBS, it is 41,495 USD/QALY, which is below the willingness-to-pay interval of 50,000-100,000 USD/QALY. Therefore, the rechargeable DBS can be considered cost-effective in the treatment of treatment-resistant obsessive–compulsive disorder, while the non-rechargeable one proves to be less cost-effective.

Finally, in refractory epilepsy, a study conducted by Chan et al. (2022) [16], described a comparison of the cost-effectiveness of DBS with the standard treatment for epilepsy. An estimated ICER of 46,640 EUR/QALY was presented for DBS, being barely more attractive than vagus nerve stimulation (47,155 EUR/QALY), but it is worth noting that DBS has a competitive price even in indications where alternative neuromodulation strategies exist.

## 2. Targets

The sensorimotor GPi and STN [17] are the main targets of DBS in PD, and several results indicate that the pedunculopontine nucleus (PPN) [18] and globus pallidus externus (GPe) [19] might be effective targets as well, while a study conducted by Drouot et al. (2004) [20] showed that the superficial motor cortex stimulation using a dural electrode could alleviate PD manifestations. For most of the dystonia, the posteroventral GPi has become preferred as a stimulation site [21]. However, the thalamus’s ventralis intermediate nucleus (Vim) remains the primary target for essential tremor [22], while STN and GPi improve the treatment of PD’s tremor [23,24].

It is important to note that differences between STN and GPi electrode placement targets exist. Some studies show that STN represents a better site than GPi for reducing the motor symptoms as well as the levodopa intake [25,26,27,28]. On the other hand, another meta-analysis showed that regarding the motor symptoms and levodopa intake, there was no difference between STN and GPi, but the main advantage of choosing the GPi was the reduced dyskinesia while on medication [29].

A study conducted by Schmidt et al. (2024) [30] on a cohort of six patients with PD showed that the dual-target DBS of both STN and globus pallidus led to improvements in motor symptoms (UPDRS-III), a reduced levodopa requirement, and an increased “on” time without the presence of dyskinesia over 2 years. Moreover, the dual-target DBS showed more than 8 h of dyskinesia absence during the “on” time, compared to approximately 4.5 h in single-target DBS [31,32].

It is also worth noting that the dentatorubrothalamic tract (DRTT) also represents an important and efficient target in the treatment of essential tremor [33,34]. A study conducted by Dembek et al. (2020) [35] showed that the efficiency of DBS between the posterior subthalamic area and Vim depends on the proximity of the lead to the DRTT, demonstrating that shorter distances to this target resulted in better clinical outcomes, even at lower amplitudes, suggesting a tractographic location rather than a simpler anatomic location.

## 3. Mechanism

A general theory of the causal therapeutic mechanism of DBS has not emerged, but the widespread effects are considered multifactorial [36]. Common theories include local suppression and informational lesion.

DBS may suppress neural activity transiently to relieve PD symptoms, as stereotactic ablation would. Lesions in GPi and STN produce comparable effects supporting functional deafferentation [37,38,39,40]. Neuronal firing suppression in the STN, GPi, and thalamus was linked to GABAergic activation, synaptic depression, or depolarization blockade [41]. Yet computational and preclinical studies suggest DBS effects go beyond local inhibition [42].

Rather than reducing local firing alone, DBS may induce an “informational lesion” by affecting pathological activity. However, the informational lesion theory and the way DBS inhibits the neuronal networks as presented in some older studies [43,44] seems to be just partially correct. Lowet et al. (2022) [45] conducted a study regarding the hippocampal DBS, which showed an increase in the amplitude of the membrane voltage depolarization, especially during the 140 Hz stimulation compared to the 40 Hz and the increased spike rates, stating that the neurons are still active. However, by using optogenetics to mimic the rhythmic theta impulses of the hippocampus, the researchers demonstrated that DBS alters information processing by making the neurons unresponsive to normal rhythmic impulses. Therefore, the DBS does not inhibit or silence the neurons; instead, it decouples the pathological rhythmic input from the output through a functional rather than a suppressive mechanism.

Moreover, besides the classical electrical mechanisms, recent studies have explored a new possibility of wireless DBS by combining optogenetics with remotely driven light sources placed deep in the brain and activated by X-rays. In these models, the neurons are rendered light-sensitive through the expression of the opsins, while particles that are delivered locally emit visible photons at the moment of X-ray exposure, therefore modulating the neuronal activity, with cellular specificity, without the need for permanent optical implants. This approach is called X-ray-mediated optogenetics, and it achieved the activation of a specific neuron population, such as the mesencephalic dopaminergic neurons, which led to behavioral modifications of the rodents, mimicking a wireless DBS action [46,47]. Recent scintillator formulations, such as Cerium-doped gadolinium aluminum gallium garnet (Ce:GAGG) particles, appear short-term biocompatible and capable of eliciting neuronal firing without significant inflammatory responses [48]. However, this method is still in a preclinical phase and more research, as well as more long-term results regarding its safety and efficacy, is needed.

DBS may also work by backpropagating electrical signals to the motor cortex. Rodent studies suggest that antidromic activation contributes to symptom relief [49,50], and invasive patient recordings show low-latency antidromic evoked potentials [51,52]. However, non-human primate studies challenge this, showing that antidromic activation is unstable and may shift to synaptic depression [53]. Since both STN and GPi-DBS are effective for PD but only STN-DBS consistently triggers antidromic activity, the clinical relevance of this mechanism is uncertain [53]. Additionally, short-latency cortical responses in GPi-DBS may result from direct corticospinal tract stimulation rather than true antidromic activation [54].

## 4. DBS Stimulation in Other Pathologies (Psychiatric Pathologies)

DBS has been proposed as a therapeutic technique in patients with treatment-resistant problems of psychiatry such as major depressive disorder and obsessive–compulsive disorder (OCD). Early results in open-label trials were encouraging [55]. Randomized controlled trials (RCTs) have produced more variable outcomes and focused on broader brain network models instead of isolated anatomical targets [56,57]. Therefore, the psychiatric conditions take place in cognitive and emotional circuits, while DBS acts mainly on distributed neural networks rather than specific and singular brain regions. Thus, the research now focuses on the patient-specific strategies, through the exploration of neurophysiological real-time biomarkers in order to achieve the best clinical outcomes, through precision medicine [58].

While single-center studies have shown promising results, large-scale studies struggle to do so, largely because therapeutic targets have not been defined or engaged. Unlike movement disorders, which show rapid and observable clinical responses to DBS, psychiatric symptoms develop slowly, and it is not possible to fine-tune stimulation parameters in real time. To circumvent this problem, recently described “closed-loop” systems adapt stimulation to individual neurophysiological markers [59]. Newer approaches now focus on patient-specific biomarkers, functional domains including cognitive control, and network-based stimulation strategies. These strategies target a transition from categorical diagnoses, such as major depressive disorder, to precision medicine models anchored in measurable brain–behavior relationships. Those methods have their scalability and regulatory challenges, but represent a significant step towards adapting DBS therapy to complex and heterogeneous psychiatric conditions.

OCD is a chronic condition characterized by intrusive thoughts and compulsive behaviors [60] affecting 2–3% of the population [61]. Cognitive behavioral therapy and SSRIs (selective serotonin reuptake inhibitors) represent standard treatments [62,63], but 25–40% remain treatment refractory and impaired [64]. Neuromodulation includes deep transcranial magnetic stimulation (dTMS), which has been FDA approved for OCD [65]. DBS proves to have the best efficacy among the neuromodulatory methods for the treatment-resistant OCD, using as the most common targets the anterior limb of the internal capsule (ALIC), the nucleus accumbens (NAc), the ventral capsule/ventral striatum (VC/VS), the subthalamic nucleus (STN), and the bed nucleus of the stria terminalis (BNST) [66,67,68]. DBS response rates of nearly 60% and symptom reductions of 40–45% have been observed using the Y-BOCS scores, therefore being approved under a Humanitarian Device Exemption since 2009 [67]. Optimal stimulation parameters and predictors of response are under research.

Schizophrenia has both positive symptoms, such as hallucinations, delusions, and disorganized thinking, and negative symptoms such as apathy, flat affect, and cognitive deficits, with a prevalence of 14.2 to 23.6 million [69]. Although repetitive TMS has been investigated, mainly for auditory hallucinations, there is little evidence of cognitive benefit from DLPFC (dorsolateral prefrontal cortex) stimulation [70]. tDCS (transcranial direct current stimulation) reduced auditory hallucinations by 31% [71], and ECT is effective for positive symptoms but generally less effective than antipsychotics alone [72]. Emerging DBS research reported improvements in positive and negative symptoms and comorbid conditions including depression and OCD [73].

Intracranial recordings represent a new modality of DBS that can be efficient in treatment-resistant depression (TRD). In this pioneering study conducted by Sheth et al. (2022) [74], the team used implemented stereo-EEG (sEEG) to map individual network dynamics and guide stimulation to two key regions: subcallosal cingulate (SCC) and VC/VS. Such a dual target strategy together with data-driven parameter selection resulted in the complete remission of depressive symptoms in the first treated patient. Notably, the personalized DBS settings were obtained by the “inverse solution method,” optimizing stimulation on the basis of electrophysiological mood state signatures.

Another pathology that could be efficiently treated using DBS is treatment-refractory anorexia nervosa. Studies have shown that targeting subcallosal cingulate (Cg25) and NAc may improve mood and anxiety, affective regulation, and quality of life [75,76]. Moreover, some patients maintained weight gain with positive metabolic and neural changes, suggesting that DBS modulated dysfunctional brain networks associated with emotional and cognitive processing related to the disorder.

## 5. Complications and Adverse Effects of the DBS

However, even if the DBS shows great benefits for a wide range of pathologies, the surgical procedure can also lead to different complications (Table 2), some of them related to the surgical technique and mechanical factors, while some are related to psychiatric exacerbations, as presented in a study by Lapa et al. (2024) [77], which analyzed the DBS effects on treatment-resistant depression patients, showing that complications manifesting as the occurrence or worsening of the depressive symptoms, anxiety, and mania occurred in 18.4%, 9.1%, and 5.1% of patients.

A study published by Tabaja et al. (2023) [78], based on a sample of 1087 patients, showed a reassuring infection rate of 5% for the primary DBS implantation surgery and only 2% for the revision surgeries, showing a positive correlation with the body mass index, diabetes mellitus, and male gender.

Hardware-related problems, including lead fractures, are fairly common, and in reported studies, the occurrence rate has ranged between 1.46% and 15.2%, which shows major consequences for clinical practice [79,80]. If a clinician suspects a hardware complication, then an organized diagnostic approach through the evaluation of the impedance, assessment of voltage-related symptoms, and radiological imaging must occur [81]. Jiang et al. (2015) [82] in their experimental study demonstrated that the mechanical durability of leads was strongly related to maintaining the helical shape to avoid fatigue failure.

Moreover, a study conducted by Mackel et al. (2020) [83] showed that 60-centimeter extension wires with parietal connectors exhibit a lower fracture rate than 40 cm extensions with postauricular or parietal connections but at increased risk of tethering.

Tethering, which is also known as bowstringing of the extension wire, has become a more recognized complication of DBS. The frequency of tethering has been reported from 0.1% to 2.6% in patients and from 0.2% to 1.3% per implanted electrode; overall, it is estimated at 0.7 cases per 100 electrode-years [79,84].

Chronic DBS can also lead to neurological side effects, which may result from unintentional electrical current propagation to eloquent areas or from tissue damage caused by the interaction between the leads and external electromagnetic sources. When the electrical field of the DBS spreads beyond the sensory motor target, the recruitment of the nearby corticospinal and corticobulbar fibers may appear, which can lead to either focal muscular contractions or dysarthria. However, if the cerebellothalamic and efferent cerebellar fibers are affected, ataxia and imbalance may occur, especially if the patient has a Vim or PSA DBS implanted for the treatment of essential tremor [85]. Similar current spillover toward limbic and associative subterritories of the STN, the internal capsule, mammillothalamic tract, or optic pathways can lead to transient mood and cognitive changes, paresthesias, diplopia, phosphenes, or tonic gaze deviation [85].

Over the years of continuous therapy, the chronic side effects observed by Morrison et al. (2021) [86] were habituation and either progressive dysarthria or ataxia, which were associated with the long-term DBS.

Therefore, in order to avoid all of these adverse effects, the technological development of directional and segmented electrodes that can guide the electrical field away from vulnerable tracts located near the implantation point, as well as the use of tractography to avoid the cerebellothalamic pathways such as DRTT, improves the control of essential tremor with fewer side effects [86].

Beyond the electrical spillage, the DBS can interact with some external electromagnetic sources such as electrocautery, MRI, cardioversion, or even certain security scanners, generating unintended currents that can temporarily stop the IPG and reset the parameters, or in some extreme cases, focal radiofrequency heating can occur at the electrode tip, with reported thalamic lesions, dysarthria, hemiparesis, or other irreversible neurological deficits [87].

Therefore, the chronic side effects of the electrical field produced by the DBS are not caused by the structural damage produced by the electrodes, but rather by the interaction of the current and the nearby circuits, taking into account the rare cases, but with important clinical implications, where an interaction between the hardware and other external electromagnetic sources appears.

All of these complications must be taken into account by the physician and the patient, as they pose both a health risk and an economic risk, since Bishay et al. (2024) [88] reported in a systematic review that the total cost of a DBS surgery could exceed USD 40.000.

**Table 2:** A summary table across five large-scale studies that shows the most common procedure- and device-related complications and their prevalence that can occur after the DBS surgery. *Abbreviations:* STN = subthalamic nucleus; GPi = globus pallidus internus; ViM = ventral intermediate nucleus of the thalamus; PSA = posterior subthalamic area; MER = micro-electrode recording; PLE = peri-lead oedema; IPG = implantable pulse generator; PD = Parkinson’s disease; ET = essential tremor; HA = headache; OCD = obsessive–compulsive disorder.

#	Study (First Author, Year)	Design/Scope	Patients (n)	DBS Location(s)	Principal Pathology/Indications	Salient Complication Findings
1	Rasiah et al. (2023) [89]	Preferred Reporting Items for Systematic Reviews and Meta-Analyses (PRISMA) meta-analysis of 262 studies	21,261	STN, GPi, ViM	Parkinson’s disease	Revision 4.9%; infection 4.2%; lead malposition 3.3%; hemorrhage 2.4% (risk rises with >1 MER track)
2	Tian et al. (2022) [90]	Meta-analysis of 10 studies	1354	STN, GPi, ViM, PSA	Predominantly PD/ET	Pooled PLE incidence 35.8%; symptomatic PLE 3.1%
3	Bullard et al. (2020) [91]	Systematic review of 240 studies	34,089	Mixed targets	Broad neurology and psychiatry indications	Infection 4.57%; IPG malfunction 3.25%; hemorrhage 2.86%; lead fracture 2.56%
4	Radziunas et al. (2018) [92]	Prospective single-center cohort	22	STN	Parkinson’s disease	31.8% developed early neuro-psychiatric events (psychosis, delirium) after STN-DBS
5	Jitkritsadakul et al. (2017) [79]	Systematic review of 96 studies	8983	Mixed (STN, GPi, ViM, etc.)	PD, dystonia, Tourette, epilepsy, cluster HA, OCD	Hardware complications overall 11.75%; infections 5.1%; lead migration 1.6%

## 6. DBS and Connectomics

DBS is one of the few neurosurgical interventions that allows clinicians to observe neuronal network behavior in real time in awake patients. By threading recording electrodes into key circuits, we can follow the moment-to-moment responses of those circuits to therapeutic pulses. Such observations do more than satisfy scientific curiosity; they clarify the pathological circuitry that drives movement disorders and illuminate how neuromodulation exerts its benefits. Grasping this link between network dynamics and clinical response is pivotal for designing the next generation of adaptive stimulators, capable of adjusting output on the fly [93]. Recent engineering advances now even allow months-long tracking of population activity during high-frequency stimulation, using signals recorded directly from the implanted leads [94,95,96].

Retrospective patient series add further insight. Analyses of individuals treated with subthalamic nucleus (STN) or pallidal DBS for PD, thalamic DBS for essential tremor, and pallidal DBS for dystonia consistently reveal the same principle: the extent to which electrodes engage specific structural and functional networks predicts symptom relief [97,98,99,100]. Notably, anatomical connectivity and physiological coupling each appear to carry independent prognostic weight, underscoring the importance of considering both when forecasting outcome or planning surgery [97].

Zooming in to the cellular scale, intraoperative single-unit recordings have exposed yet another layer of complexity. High-frequency pulses can induce synaptic depression, which briefly silences target neurons, resulting in fleeting “silent periods” in their firing. These pauses correlate strongly with clinical benefit in PD [101,102].

Complementing these empirical findings is a recently developed integrative computational model that reproduces the firing rate changes observed in the STN, substantia nigra pars reticulata, globus pallidus internus, and the thalamic ventral intermediate nucleus across a wide range of stimulation frequencies [101,102,103,104]. The model starts from a straightforward premise: every DBS pulse activates the presynaptic boutons of all afferent axons terminating in the target nucleus [105]. Whether the net effect is excitation, inhibition, or a mix of both depends on the spatial pattern and balance of converging excitatory and inhibitory inputs [106].

## 7. DBS and AI

More generally, the systems developed are considered artificial intelligence (AI), as they simulate or emulate some aspects of human intelligence. AI-driven tools can analyze, contextualize data, and find new insights from existing datasets independently. In medicine, AI is applied to image analysis, patient care, clinical decision support, disease detection, and drug development [107]. A major branch of AI is machine learning (ML), where computers find patterns and enhance predictions with experience. The deep learning subset of ML uses artificial neural networks to model relationships in data [108]. ML techniques may be supervised or unsupervised learning. Supervised learning trains models on labeled data via neural networks, support vector machines, and random forests [109,110] while unsupervised learning detects structures in unlabeled data via K-means clustering, PCA, and Gaussian mixture models.

ML has great promise for supporting clinical decision making in neurosurgery. Systematic reviews by Celtikci et al. (2018) [111] as well as Buchlak et al. (2020) [112] highlighted how ML can handle highly complex and high-dimensional data better than traditional statistical methods. Another study [113,114] also underlined that ML can be applied to neurosurgical care and that it can outperform human experts in diagnosis, planning, and outcome prediction, while avoiding publication bias. However, such reviews have not directly focused on the application of ML in DBS.

AI is increasingly applied to DBS research in order to increase treatment precision and/or adaptability. AI models, especially those based on machine learning, have been applied to patient classification, treatment outcome prediction, and personalization of the stimulation parameters. In DBS, AI enables closed-loop systems to adjust stimulation in real time according to neural signals. ML has been applied to various domains of DBS such as patient selection, surgical targeting, stimulation programming, and mechanisms [115]. Analyzing large-scale datasets, including neuroimaging, electrophysiological signals, and wearable sensors, enables ML models to perform personalized treatment planning and real-time adaptive stimulation. All these approaches may help with improving precision, or with reducing trial and error when adjusting therapy, or with improving patient outcomes from a data-driven decision support perspective; yet a major challenge is still the interpretability of such models. Newer efforts in explainable AI (XAI) seek to make model decisions transparent to increase clinical trust and informed decision making in neuromodulation [116].

ML techniques are increasingly applied to develop and optimize DBS systems. For example, Jovanov et al. (2018) [117] used genetic algorithms to optimize treatment on a real-time basis, presenting a new adaptive DBS hardware platform. In another hardware-related challenges study, Zhu et al. (2020) [118] investigated the performance and resource efficiency of seizure detection for PD using EEG/ECoG signals with a custom decision tree algorithm. In a similar manner, De La Pava et al. (2016) [119] applied KNN models to tissue-activation visualization for DBS modeling. ML has also been useful during surgical planning in addition to being hardware. Through the interpretation of intraoperative signals (microelectrode recordings (MERs)), ML assists with anatomical targeting and electrode placement strategies. Though many studies use MERs for classification and localization, few have shown robust correlations with clinical outcomes. However, anatomical modeling using imaging data based on ML is developing with a potential for better accuracy and flexibility than classical direct or indirect targeting methods [120,121]. Despite the growing application of ML in DBS, the evidence thus far demonstrates great potential in several aspects of therapy ranging from candidate selection to programming optimization. The focus on interpretable models (explainable AI) is key to clinical integration: clinicians need to understand and trust algorithmic decisions. Beyond these, nascent work to characterize genetic profiles and Parkinson’s-associated mutations may individualize DBS treatment and forecast therapeutic efficacy [122,123].

A recent study conducted by Ferrea et al. (2024) [124] used explainable machine learning to explore variability in quality-of-life outcomes after DBS in the PD population. Incorporating demographic, clinical, and neuroimaging data along with neurophysiological parameters, the model predicted postoperative QoL change and preoperative PDQ-39 scores, as well as upper beta band activity > 20 Hz in STN. Additionally, vertical positioning of electrode contact relative to the z = −7 coordinate of standard mNI space impacted outcomes, with much better placements related to better QoL. They indicate that AI could considerably improve patient stratification, surgical planning, and personalized DBS therapy.

Recent advances suggest that machine learning may enable the prediction of patient-specific sensory outcomes to help personalize DBS. A predictive model that predicts the paresthesia occurrence as well as the somatic location of it, based on stimulation parameters and volume of tissue activation (VTA) metrics, has been developed by Halasz et al. (2024) [125]. Their results suggest the utility of computational models for DBS programming, potentially reducing trial-and-error adjustments and improving treatment efficiency. Predictions of paresthesia presence were robust, but the accuracy of somatic localization decreased in unseen cases, highlighting the need for personalized data for accurate sensory mapping. These insights help optimize current clinical workflows and pave the way for coupling DBS to computer–brain interfaces based on targeted sensory feedback.

## 8. Conclusions

DBS has evolved from being a treatment for complicated motor fluctuations in people with PD who are not responsive to levodopa into a versatile neurosurgical platform for the treatment of disorders across the motor–psychiatric spectrum. Studies have shown that, despite eligibility requirements, DBS offers favorable cost-effectiveness and sometimes even net savings compared to the best medical therapy for PD. The procedure now extends therapeutically beyond the canonical subthalamic nucleus and globus pallidus internus targets to include ventral thalamic, posterior subthalamic, and corticostriatal targets, targeting specific symptom networks. While we do not yet have a clear action mechanism that leads to the beneficial effects of DBS, various lines of converging evidence suggest a model of action that involves modulating the dynamics of distributed neural circuits rather than simply inhibiting localized neuronal activity. Moreover, connectomic mapping, as well as machine learning, will improve the DBS functionality to make it more adaptable to each patient’s complex neural circuits. Taken together, the evidence presented in this review underscores the role of DBS as a cornerstone of precision neurotherapeutics, offering sustained clinical benefits and increasingly supported by economic and technological rationales.

## Figures and Tables

**Table 1:** Indications for DBS surgery according to CAPSIT-PD [10].

Criteria	Requirement
Diagnosis	Confirmed Idiopathic Parkinson’s Disease (IPD) with at least 5 years of disease duration.
Dopaminergic Responsiveness	A pharmacologic test with L-dopa or apomorphine should show at least a 33% decrease in the Unified Parkinson’s Disease Rating Scale (UPDRS) Part III score.
Motor Symptoms	Severe motor fluctuations, dyskinesias, or medication-resistant tremors significantly impair quality of life.
Cognitive and Behavioral Status	No severe cognitive impairment or dementia (Mattis Dementia Rating Scale cut-off: 130 or 120).
Psychiatric Exclusion Criteria	No severe psychiatric disorders, particularly major depression (Montgomery-Asberg Depression Rating Scale (MADRS): 7–19 cut-off) or active psychosis.
Medication Stability	Stable antiparkinsonian medication for at least 3 months preoperatively.
Age Consideration	No strict age limit, but younger patients tend to respond better; caution in elderly patients with cognitive risk.
Imaging Exclusion	No structural brain abnormalities suggestive of atypical parkinsonism (MRI recommended).
Functional Impairment	Significant functional disability despite optimal medical therapy

## Data Availability

The data presented in this study are available on request from the corresponding author.

## References

[1] Emamzadeh F.N., Surguchov A. (2018). Parkinson’s Disease: Biomarkers, Treatment, and Risk Factors. Front. Neurosci..

[2] Balestrino R., Schapira A.H.V. (2020). Parkinson disease. Eur. J. Neurol..

[3] Tanner C.M., Ostrem J.L. (2024). Parkinson’s Disease. N. Engl. J. Med..

[4] Lozano A.M., Lipsman N., Bergman H., Brown P., Chabardes S., Chang J.W., Matthews K., McIntyre C.C., Schlaepfer T.E., Schulder M. (2019). Deep brain stimulation: Current challenges and future directions. Nat. Rev. Neurol..

[5] Mahlknecht P., Foltynie T., Limousin P., Poewe W. (2022). How Does Deep Brain Stimulation Change the Course of Parkinson’s Disease?. Mov. Disord. Off. J. Mov. Disord. Soc..

[6] Hartmann C.J., Fliegen S., Groiss S.J., Wojtecki L., Schnitzler A. (2019). An update on best practice of deep brain stimulation in Parkinson’s disease. Ther. Adv. Neurol. Disord..

[7] Mahajan A., Butala A., Okun M.S., Mari Z., Mills K.A. (2021). Global Variability in Deep Brain Stimulation Practices for Parkinson’s Disease. Front. Hum. Neurosci..

[8] Bove F., Mulas D., Cavallieri F., Castrioto A., Chabardès S., Meoni S., Schmitt E., Bichon A., Di Stasio E., Kistner A. (2021). Long-term Outcomes (15 Years) After Subthalamic Nucleus Deep Brain Stimulation in Patients with Parkinson Disease. Neurology.

[9] Gessani A., Cavallieri F., Fioravanti V., Campanini I., Merlo A., Di Rauso G., Damiano B., Scaltriti S., Bardi E., Corni M.G. (2023). Long-term effects of subthalamic nucleus deep brain stimulation on speech in Parkinson’s disease. Sci. Rep..

[10] Defer G.L., Widner H., Marié R.M., Rémy P., Levivier M. (1999). Core assessment program for surgical interventional therapies in Parkinson’s disease (CAPSIT-PD). Mov. Disord. Off. J. Mov. Disord. Soc..

[11] Morgante L., Morgante F., Moro E., Epifanio A., Girlanda P., Ragonese P., Antonini A., Barone P., Bonuccelli U., Contarino M.F. (2007). How many parkinsonian patients are suitable candidates for deep brain stimulation of subthalamic nucleus? Results of a questionnaire. Park. Relat. Disord..

[12] Lannon M., Duda T., Mastrolonardo A., Huang E., Martyniuk A., Farrokhyar F., Xie F., Bhandari M., Kalia S.K., Sharma S. (2024). Economic Evaluations Comparing Deep Brain Stimulation to Best Medical Therapy for Movement Disorders: A Meta-Analysis. PharmacoEconomics.

[13] Nyholm D., Eggington S., Holm A. (2025). Therapies for Advanced Parkinson’s Disease in Sweden: A Cost-Effectiveness Analysis Using Real-World Data. Neurol. Ther..

[14] Kabotyanski K.E., Najera R.A., Banks G.P., Sharma H., Provenza N.R., Hayden B.Y., Mathew S.J., Sheth S.A. (2024). Cost-effectiveness and threshold analysis of deep brain stimulation vs. treatment-as-usual for treatment-resistant depression. Transl. Psychiatry.

[15] Najera R.A., Kabotyanski K.E., McLaughlin N.C., Gregory S.T., Anand A., Shofty B., Provenza N.R., Storch E.A., Goodman W.K., Sheth S.A. (2025). Cost-effectiveness analysis of deep brain stimulation versus treatment as usual for treatment-resistant obsessive-compulsive disorder. J. Neurosurg..

[16] Chan H.Y., Wijnen B.F.M., Majoie M.H.J.M., Evers S.M.A.A., Hiligsmann M. (2022). Economic evaluation of deep brain stimulation compared with vagus nerve stimulation and usual care for patients with refractory epilepsy: A lifetime decision analytic model. Epilepsia.

[17] (2001). Deep-Brain Stimulation of the Subthalamic Nucleus or the Pars Interna of the Globus Pallidus in Parkinson’s Disease. N. Engl. J. Med..

[18] Stefani A., Lozano A.M., Peppe A., Stanzione P., Galati S., Tropepi D., Pierantozzi M., Brusa L., Scarnati E., Mazzone P. (2007). Bilateral deep brain stimulation of the pedunculopontine and subthalamic nuclei in severe Parkinson’s disease. Brain.

[19] Vitek J.L., Hashimoto T., Peoples J., DeLong M.R., Bakay R.A.E. (2004). Acute stimulation in the external segment of the globus pallidus improves parkinsonian motor signs. Mov. Disord..

[20] Drouot X., Oshino S., Jarraya B., Besret L., Kishima H., Remy P., Dauguet J., Lefaucheur J.P., Dollé F., Condé F. (2004). Functional Recovery in a Primate Model of Parkinson’s Disease following Motor Cortex Stimulation. Neuron.

[21] Andrews L., Ali A.M.S., Elmolla M., Keller S.S., Bhojak M., Osman-Farah J., Macerollo A. (2024). Directional deep brain stimulation for cervical dystonia: Outcomes, challenges and future directions. Deep Brain Stimul..

[22] Guehl D., Guillaud E., Langbour N., Doat E., Auzou N., Courtin E., Branchard O., Engelhardt J., Benazzouz A., Eusebio A. (2023). Usefulness of thalamic beta activity for closed-loop therapy in essential tremor. Sci. Rep..

[23] Stover N.P., Okun M.S., Evatt M.L., Raju D.V., Bakay R.A.E., Vitek J.L. (2005). Stimulation of the Subthalamic Nucleus in a Patient with Parkinson Disease and Essential Tremor. Arch. Neurol..

[24] Diamond A., Shahed J., Jankovic J. (2007). The effects of subthalamic nucleus deep brain stimulation on parkinsonian tremor. J. Neurol. Sci..

[25] Lachenmayer M.L., Mürset M., Antih N., Debove I., Muellner J., Bompart M., Schlaeppi J.-A., Nowacki A., You H., Michelis J.P. (2021). Subthalamic and pallidal deep brain stimulation for Parkinson’s disease—Meta-analysis of outcomes. Npj Park. Dis..

[26] Fan S., Liu D., Shi L., Meng F., Fang H., Liu H., Zhang H., Yang A., Zhang J. (2022). Differential Effects of Subthalamic Nucleus and Globus Pallidus Internus Deep Brain Stimulation on Motor Subtypes in Parkinson’s Disease. World Neurosurg..

[27] Huhn M., Prewett M., Rossignol J., Dunbar G.L. (2024). Comparison of the Long-Term Efficacy of Targeting the Subthalamic Nucleus Versus the Globus Pallidus Interna for Deep Brain Stimulation Treatment of Motor Dysfunction in Patients with Parkinson’s Disease: A Meta-Analysis Study. Park. Dis..

[28] Rajamani N., Friedrich H., Butenko K., Dembek T., Lange F., Navrátil P., Zvarova P., Hollunder B., De Bie R.M.A., Odekerken V.J.J. (2024). Deep brain stimulation of symptom-specific networks in Parkinson’s disease. Nat. Commun..

[29] Zhang J., Li J., Chen F., Liu X., Jiang C., Hu X., Ma L., Xu Z. (2021). STN versus GPi deep brain stimulation for dyskinesia improvement in advanced Parkinson’s disease: A meta-analysis of randomized controlled trials. Clin. Neurol. Neurosurg..

[30] Schmidt S.L., Chowdhury A.H., Mitchell K.T., Peters J.J., Gao Q., Lee H.-J., Genty K., Chow S.-C., Grill W.M., Pajic M. (2024). At home adaptive dual target deep brain stimulation in Parkinson’s disease with proportional control. Brain.

[31] Deuschl G., Schade-Brittinger C., Krack P., Volkmann J., Schäfer H., Bötzel K., Daniels C., Deutschländer A., Dillmann U., Eisner W. (2006). A Randomized Trial of Deep-Brain Stimulation for Parkinson’s Disease. N. Engl. J. Med..

[32] Weaver F.M. (2009). Bilateral Deep Brain Stimulation vs Best Medical Therapy for Patients with Advanced Parkinson Disease A Randomized Controlled Trial. JAMA.

[33] Fenoy A.J., Schiess M.C. (2017). Deep Brain Stimulation of the Dentato-Rubro-Thalamic Tract: Outcomes of Direct Targeting for Tremor. Neuromodul. Technol. Neural Interface.

[34] Chazen J.L., Sarva H., Stieg P.E., Min R.J., Ballon D.J., Pryor K.O., Riegelhaupt P.M., Kaplitt M.G. (2018). Clinical improvement associated with targeted interruption of the cerebellothalamic tract following MR-guided focused ultrasound for essential tremor. J. Neurosurg..

[35] Dembek T.A., Petry-Schmelzer J.N., Reker P., Wirths J., Hamacher S., Steffen J., Dafsari H.S., Hövels M., Fink G.R., Visser-Vandewalle V. (2020). PSA and VIM DBS efficiency in essential tremor depends on distance to the dentatorubrothalamic tract. NeuroImage Clin..

[36] Krauss J.K., Lipsman N., Aziz T., Boutet A., Brown P., Chang J.W., Davidson B., Grill W.M., Hariz M.I., Horn A. (2021). Technology of deep brain stimulation: Current status and future directions. Nat. Rev. Neurol..

[37] Alvarez L., Macias R., Pavón N., López G., Rodríguez-Oroz M.C., Rodríguez R., Alvarez M., Pedroso I., Teijeiro J., Fernández R. (2009). Therapeutic efficacy of unilateral subthalamotomy in Parkinson’s disease: Results in 89 patients followed for up to 36 months. J. Neurol. Neurosurg. Psychiatry.

[38] Rodriguez-Rojas R., Pineda-Pardo J.A., Martinez-Fernandez R., Kogan R.V., Sanchez-Catasus C.A., Del Alamo M., Hernández F., García-Cañamaque L., Leenders K.L., Obeso J.A. (2020). Functional impact of subthalamotomy by magnetic resonance-guided focused ultrasound in Parkinson’s disease: A hybrid PET/MR study of resting-state brain metabolism. Eur. J. Nucl. Med. Mol. Imaging.

[39] Martínez-Fernández R., Máñez-Miró J.U., Rodríguez-Rojas R., Del Álamo M., Shah B.B., Hernández-Fernández F., Pineda-Pardo J.A., Monje M.H.G., Fernández-Rodríguez B., Sperling S.A. (2020). Randomized Trial of Focused Ultrasound Subthalamotomy for Parkinson’s Disease. N. Engl. J. Med..

[40] Benazzouz A., Hallett M. (2000). Mechanism of action of deep brain stimulation. Neurology.

[41] Davidson B., Milosevic L., Kondrataviciute L., Kalia L.V., Kalia S.K. (2024). Neuroscience fundamentals relevant to neuromodulation: Neurobiology of deep brain stimulation in Parkinson’s disease. Neurotherapeutics.

[42] Anderson R.W., Farokhniaee A., Gunalan K., Howell B., McIntyre C.C. (2018). Action potential initiation, propagation, and cortical invasion in the hyperdirect pathway during subthalamic deep brain stimulation. Brain Stimulat..

[43] Bourne S.K., Eckhardt C.A., Sheth S.A., Eskandar E.N. (2012). Mechanisms of deep brain stimulation for obsessive compulsive disorder: Effects upon cells and circuits. Front. Integr. Neurosci..

[44] Grill W.M., Snyder A.N., Miocinovic S. (2004). Deep brain stimulation creates an informational lesion of the stimulated nucleus. Neuroreport.

[45] Lowet E., Kondabolu K., Zhou S., Mount R.A., Wang Y., Ravasio C.R., Han X. (2022). Deep brain stimulation creates informational lesion through membrane depolarization in mouse hippocampus. Nat. Commun..

[46] Chen Z., Tsytsarev V., Finfrock Y.Z., Antipova O.A., Cai Z., Arakawa H., Lischka F.W., Hooks B.M., Wilton R., Wang D. (2021). Wireless Optogenetic Modulation of Cortical Neurons Enabled by Radioluminescent Nanoparticles. ACS Nano.

[47] Matsubara T., Yanagida T., Kawaguchi N., Nakano T., Yoshimoto J., Sezaki M., Takizawa H., Tsunoda S.P., Horigane S., Ueda S. (2021). Remote control of neural function by X-ray-induced scintillation. Nat. Commun..

[48] Hildebrandt M., Koshimizu M., Asada Y., Fukumitsu K., Ohkuma M., Sang N., Nakano T., Kunikata T., Okazaki K., Kawaguchi N. (2024). Comparative Validation of Scintillator Materials for X-Ray-Mediated Neuronal Control in the Deep Brain. Int. J. Mol. Sci..

[49] Li Q., Ke Y., Chan D.C.W., Qian Z.-M., Yung K.K.L., Ko H., Arbuthnott G.W., Yung W.-H. (2012). Therapeutic deep brain stimulation in Parkinsonian rats directly influences motor cortex. Neuron.

[50] Sanders T.H., Jaeger D. (2016). Optogenetic stimulation of cortico-subthalamic projections is sufficient to ameliorate bradykinesia in 6-ohda lesioned mice. Neurobiol. Dis..

[51] Chen W., de Hemptinne C., Miller A.M., Leibbrand M., Little S.J., Lim D.A., Larson P.S., Starr P.A. (2020). Prefrontal-Subthalamic Hyperdirect Pathway Modulates Movement Inhibition in Humans. Neuron.

[52] Jorge A., Lipski W.J., Wang D., Crammond D.J., Turner R.S., Richardson R.M. (2022). Hyperdirect connectivity of opercular speech network to the subthalamic nucleus. Cell Rep..

[53] Johnson L.A., Wang J., Nebeck S.D., Zhang J., Johnson M.D., Vitek J.L. (2020). Direct Activation of Primary Motor Cortex during Subthalamic But Not Pallidal Deep Brain Stimulation. J. Neurosci. Off. J. Soc. Neurosci..

[54] Miocinovic S., de Hemptinne C., Chen W., Isbaine F., Willie J.T., Ostrem J.L., Starr P.A. (2018). Cortical Potentials Evoked by Subthalamic Stimulation Demonstrate a Short Latency Hyperdirect Pathway in Humans. J. Neurosci. Off. J. Soc. Neurosci..

[55] Holtzheimer P.E., Husain M.M., Lisanby S.H., Taylor S.F., Whitworth L.A., McClintock S., Slavin K.V., Berman J., McKhann G.M., Patil P.G. (2017). Subcallosal cingulate deep brain stimulation for treatment-resistant depression: A multisite, randomised, sham-controlled trial. Lancet Psychiatry.

[56] Raymaekers S., Luyten L., Bervoets C., Gabriëls L., Nuttin B. (2017). Deep brain stimulation for treatment-resistant major depressive disorder: A comparison of two targets and long-term follow-up. Transl. Psychiatry.

[57] Pycroft L., Stein J., Aziz T. (2018). Deep brain stimulation: An overview of history, methods, and future developments. Brain Neurosci. Adv..

[58] Alagapan S., Choi K.S., Heisig S., Riva-Posse P., Crowell A., Tiruvadi V., Obatusin M., Veerakumar A., Waters A.C., Gross R.E. (2023). Cingulate dynamics track depression recovery with deep brain stimulation. Nature.

[59] Widge A.S. (2024). Closing the loop in psychiatric deep brain stimulation: Physiology, psychometrics, and plasticity. Neuropsychopharmacology.

[60] Vahia V.N. (2013). Diagnostic and Statistical Manual of Mental Disorders: DSM-5.

[61] Strom N.I., Soda T., Mathews C.A., Davis L.K. (2021). A dimensional perspective on the genetics of obsessive-compulsive disorder. Transl. Psychiatry.

[62] Koran L.M., Hanna G.L., Hollander E., Nestadt G., Simpson H.B. (2007). American Psychiatric Association Practice guideline for the treatment of patients with obsessive-compulsive disorder. Am. J. Psychiatry.

[63] Romanelli R.J., Wu F.M., Gamba R., Mojtabai R., Segal J.B. (2014). Behavioral therapy and serotonin reuptake inhibitor pharmacotherapy in the treatment of obsessive-compulsive disorder: A systematic review and meta-analysis of head-to-head randomized controlled trials: Review: Behavioral Therapy and SRIs in the Treatment of OCD. Depress. Anxiety.

[64] Fineberg N.A., Brown A., Reghunandanan S., Pampaloni I. (2012). Evidence-based pharmacotherapy of obsessive-compulsive disorder. Int. J. Neuropsychopharmacol..

[65] Carmi L., Tendler A., Bystritsky A., Hollander E., Blumberger D.M., Daskalakis J., Ward H., Lapidus K., Goodman W., Casuto L. (2019). Efficacy and Safety of Deep Transcranial Magnetic Stimulation for Obsessive-Compulsive Disorder: A Prospective Multicenter Randomized Double-Blind Placebo-Controlled Trial. Am. J. Psychiatry.

[66] Goodman W.K., Storch E.A., Cohn J.F., Sheth S.A. (2020). Deep Brain Stimulation for Intractable Obsessive-Compulsive Disorder: Progress and Opportunities. Am. J. Psychiatry.

[67] Hageman S.B., Van Rooijen G., Bergfeld I.O., Schirmbeck F., De Koning P., Schuurman P.R., Denys D. (2021). Deep brain stimulation versus ablative surgery for treatment-refractory obsessive-compulsive disorder: A meta-analysis. Acta Psychiatr. Scand..

[68] Allam A.K., Giridharan N., Hasen M., Banks G.P., Reyes G., Dang H., Kabotyanski K.E., Hertz A.G., Heilbronner S.R., Provenza N. (2024). Effective deep brain stimulation for obsessive-compulsive disorder after failed anterior capsulotomy: Illustrative cases. J. Neurosurg. Case Lessons.

[69] Solmi M., Seitidis G., Mavridis D., Correll C.U., Dragioti E., Guimond S., Tuominen L., Dargél A., Carvalho A.F., Fornaro M. (2023). Incidence, prevalence, and global burden of schizophrenia—Data, with critical appraisal, from the Global Burden of Disease (GBD) 2019. Mol. Psychiatry.

[70] Marzouk T., Winkelbeiner S., Azizi H., Malhotra A.K., Homan P. (2020). Transcranial Magnetic Stimulation for Positive Symptoms in Schizophrenia: A Systematic Review. Neuropsychobiology.

[71] Brunelin J., Mondino M., Gassab L., Haesebaert F., Gaha L., Suaud-Chagny M.-F., Saoud M., Mechri A., Poulet E. (2012). Examining Transcranial Direct-Current Stimulation (tDCS) as a Treatment for Hallucinations in Schizophrenia. Am. J. Psychiatry.

[72] Dokucu M.E. (2015). Neuromodulation Treatments for Schizophrenia. Curr. Treat. Options Psychiatry.

[73] Plewnia C., Schober F., Rilk A., Buchkremer G., Reimold M., Wächter T., Breit S., Weiss D., Krüger R., Freudenstein D. (2008). Sustained improvement of obsessive-compulsive disorder by deep brain stimulation in a woman with residual schizophrenia. Int. J. Neuropsychopharmacol..

[74] Sheth S.A., Bijanki K.R., Metzger B., Allawala A., Pirtle V., Adkinson J.A., Myers J., Mathura R.K., Oswalt D., Tsolaki E. (2022). Deep Brain Stimulation for Depression Informed by Intracranial Recordings. Biol. Psychiatry.

[75] Lipsman N., Lam E., Volpini M., Sutandar K., Twose R., Giacobbe P., Sodums D.J., Smith G.S., Woodside D.B., Lozano A.M. (2017). Deep brain stimulation of the subcallosal cingulate for treatment-refractory anorexia nervosa: 1 year follow-up of an open-label trial. Lancet Psychiatry.

[76] Zhang H.-W., Li D.-Y., Zhao J., Guan Y.-H., Sun B.-M., Zuo C.-T. (2013). Metabolic imaging of deep brain stimulation in anorexia nervosa: A 18F-FDG PET/CT study. Clin. Nucl. Med..

[77] Lapa J.D.S., Duarte J.F.S., Campos A.C.P., Davidson B., Nestor S.M., Rabin J.S., Giacobbe P., Lipsman N., Hamani C. (2024). Adverse Effects of Deep Brain Stimulation for Treatment-Resistant Depression: A Scoping Review. Neurosurgery.

[78] Tabaja H., Yuen J., Tai D.B.G., Campioli C.C., Chesdachai S., DeSimone D.C., Hassan A., Klassen B.T., Miller K.J., Lee K.H. (2023). Deep Brain Stimulator Device Infection: The Mayo Clinic Rochester Experience. Open Forum Infect. Dis..

[79] Jitkritsadakul O., Bhidayasiri R., Kalia S.K., Hodaie M., Lozano A.M., Fasano A. (2017). Systematic review of hardware-related complications of Deep Brain Stimulation: Do new indications pose an increased risk?. Brain Stimulat..

[80] Hamani C., Lozano A.M. (2006). Hardware-Related Complications of Deep Brain Stimulation: A Review of the Published Literature. Stereotact. Funct. Neurosurg..

[81] Fernández F.S., Alvarez Vega M.A., Antuña Ramos A., Fernández González F., Lozano Aragoneses B. (2010). Lead Fractures in Deep Brain Stimulation during Long-Term Follow-Up. Park. Dis..

[82] Jiang C., Mo X., Dong Y., Meng F., Hao H., Zhang J., Feng X., Li L. (2015). An Experimental Study of Deep Brain Stimulation Lead Fracture: Possible Fatigue Mechanisms and Prevention Approach. Neuromodul. Technol. Neural Interface.

[83] Mackel C.E., Papavassiliou E., Alterman R.L. (2020). Risk Factors for Wire Fracture or Tethering in Deep Brain Stimulation: A 15-Year Experience. Oper. Neurosurg. Hagerstown Md.

[84] Fernández-Pajarín G., Sesar A., Ares B., Relova J.L., Arán E., Gelabert-González M., Castro A. (2017). Delayed complications of deep brain stimulation: 16-year experience in 249 patients. Acta Neurochir..

[85] Ineichen C., Shepherd N.R., Sürücü O. (2018). Understanding the Effects and Adverse Reactions of Deep Brain Stimulation: Is It Time for a Paradigm Shift Toward a Focus on Heterogenous Biophysical Tissue Properties Instead of Electrode Design Only?. Front. Hum. Neurosci..

[86] Morrison M.A., Lee A.T., Martin A.J., Dietiker C., Brown E.G., Wang D.D. (2021). DBS targeting for essential tremor using intersectional dentato-rubro-thalamic tractography and direct proton density visualization of the VIM: Technical note on 2 cases. J. Neurosurg..

[87] Rahimpour S., Kiyani M., Hodges S.E., Turner D.A. (2021). Deep brain stimulation and electromagnetic interference. Clin. Neurol. Neurosurg..

[88] Bishay A.E., Lyons A.T., Koester S.W., Paulo D.L., Liles C., Dambrino R.J., Feldman M.J., Ball T.J., Bick S.K., Englot D.J. (2024). Global Economic Evaluation of the Reported Costs of Deep Brain Stimulation. Stereotact. Funct. Neurosurg..

[89] Rasiah N.P., Maheshwary R., Kwon C.-S., Bloomstein J.D., Girgis F. (2023). Complications of Deep Brain Stimulation for Parkinson Disease and Relationship between Micro-electrode tracks and hemorrhage: Systematic Review and Meta-Analysis. World Neurosurg..

[90] Tian Y., Wang J., Jiang L., Feng Z., Shi X., Hao Y. (2022). The need to be alert to complications of peri-lead cerebral edema caused by deep brain stimulation implantation: A systematic literature review and meta-analysis study. CNS Neurosci. Ther..

[91] Bullard A.J., Hutchison B.C., Lee J., Chestek C.A., Patil P.G. (2020). Estimating Risk for Future Intracranial, Fully Implanted, Modular Neuroprosthetic Systems: A Systematic Review of Hardware Complications in Clinical Deep Brain Stimulation and Experimental Human Intracortical Arrays. Neuromodul. Technol. Neural Interface.

[92] Radziunas A., Deltuva V.P., Tamasauskas A., Gleizniene R., Pranckeviciene A., Surkiene D., Bunevicius A. (2020). Neuropsychiatric complications and neuroimaging characteristics after deep brain stimulation surgery for Parkinson’s disease. Brain Imaging Behav..

[93] Kühn A.A., Volkmann J. (2017). Innovations in deep brain stimulation methodology. Mov. Disord..

[94] Kehnemouyi Y.M., Wilkins K.B., Anidi C.M., Anderson R.W., Afzal M.F., Bronte-Stewart H.M. (2021). Modulation of beta bursts in subthalamic sensorimotor circuits predicts improvement in bradykinesia. Brain J. Neurol..

[95] Gilron R., Little S., Perrone R., Wilt R., De Hemptinne C., Yaroshinsky M.S., Racine C.A., Wang S.S., Ostrem J.L., Larson P.S. (2021). Long-term wireless streaming of neural recordings for circuit discovery and adaptive stimulation in individuals with Parkinson’s disease. Nat. Biotechnol..

[96] Swann N.C., De Hemptinne C., Miocinovic S., Qasim S., Ostrem J.L., Galifianakis N.B., Luciano M.S., Wang S.S., Ziman N., Taylor R. (2018). Chronic multisite brain recordings from a totally implantable bidirectional neural interface: Experience in 5 patients with Parkinson’s disease. J. Neurosurg..

[97] Horn A., Reich M., Vorwerk J., Li N., Wenzel G., Fang Q., Schmitz-Hübsch T., Nickl R., Kupsch A., Volkmann J. (2017). Connectivity Predicts deep brain stimulation outcome in P arkinson disease. Ann. Neurol..

[98] Sobesky L., Goede L., Odekerken V.J.J., Wang Q., Li N., Neudorfer C., Rajamani N., Al-Fatly B., Reich M., Volkmann J. (2022). Subthalamic and pallidal deep brain stimulation: Are we modulating the same network?. Brain.

[99] Al-Fatly B., Ewert S., Kübler D., Kroneberg D., Horn A., Kühn A.A. (2019). Connectivity profile of thalamic deep brain stimulation to effectively treat essential tremor. Brain.

[100] Okromelidze L., Tsuboi T., Eisinger R.S., Burns M.R., Charbel M., Rana M., Grewal S.S., Lu C.-Q., Almeida L., Foote K.D. (2020). Functional and Structural Connectivity Patterns Associated with Clinical Outcomes in Deep Brain Stimulation of the Globus Pallidus Internus for Generalized Dystonia. Am. J. Neuroradiol..

[101] Milosevic L., Kalia S.K., Hodaie M., Lozano A., Popovic M.R., Hutchison W. (2019). Subthalamic suppression defines therapeutic threshold of deep brain stimulation in Parkinson’s disease. J. Neurol. Neurosurg. Psychiatry.

[102] Milosevic L., Kalia S.K., Hodaie M., Lozano A.M., Fasano A., Popovic M.R., Hutchison W.D. (2018). Neuronal inhibition and synaptic plasticity of basal ganglia neurons in Parkinson’s disease. Brain J. Neurol..

[103] Milosevic L., Kalia S.K., Hodaie M., Lozano A.M., Popovic M.R., Hutchison W.D., Lankarany M. (2021). A theoretical framework for the site-specific and frequency-dependent neuronal effects of deep brain stimulation. Brain Stimulat..

[104] Milosevic L., Kalia S.K., Hodaie M., Lozano A.M., Popovic M.R., Hutchison W.D. (2018). Physiological mechanisms of thalamic ventral intermediate nucleus stimulation for tremor suppression. Brain J. Neurol..

[105] Bower K.L., McIntyre C.C. (2020). Deep brain stimulation of terminating axons. Brain Stimulat..

[106] McIntyre C.C., Grill W.M., Sherman D.L., Thakor N.V. (2004). Cellular effects of deep brain stimulation: Model-based analysis of activation and inhibition. J. Neurophysiol..

[107] Fahle S., Prinz C., Kuhlenkötter B. (2020). Systematic review on machine learning (ML) methods for manufacturing processes—Identifying artificial intelligence (AI) methods for field application. Procedia CIRP.

[108] Lavallin A., Downs J.A. (2021). Machine learning in geography–Past, present, and future. Geogr. Compass.

[109] Awad M., Khanna R. (2015). Efficient Learning Machines: Theories, Concepts, and Applications for Engineers and System Designers.

[110] Makridakis S., Spiliotis E., Assimakopoulos V. (2018). Statistical and Machine Learning forecasting methods: Concerns and ways forward. PLoS ONE.

[111] Celtikci E. (2018). A Systematic Review on Machine Learning in Neurosurgery: The Future of Decision-Making in Patient Care. Turk. Neurosurg..

[112] Buchlak Q.D., Esmaili N., Leveque J.-C., Farrokhi F., Bennett C., Piccardi M., Sethi R.K. (2020). Machine learning applications to clinical decision support in neurosurgery: An artificial intelligence augmented systematic review. Neurosurg. Rev..

[113] Senders J.T., Arnaout O., Karhade A.V., Dasenbrock H.H., Gormley W.B., Broekman M.L., Smith T.R. (2018). Natural and Artificial Intelligence in Neurosurgery: A Systematic Review. Neurosurgery.

[114] Senders J.T., Zaki M.M., Karhade A.V., Chang B., Gormley W.B., Broekman M.L., Smith T.R., Arnaout O. (2018). An introduction and overview of machine learning in neurosurgical care. Acta Neurochir..

[115] Stanslaski S., Summers R.L.S., Tonder L., Tan Y., Case M., Raike R.S., Morelli N., Herrington T.M., Beudel M., Ostrem J.L. (2024). Sensing data and methodology from the Adaptive DBS Algorithm for Personalized Therapy in Parkinson’s Disease (ADAPT-PD) clinical trial. Npj Park. Dis..

[116] Allen B. (2023). Discovering Themes in Deep Brain Stimulation Research Using Explainable Artificial Intelligence. Biomedicines.

[117] Jovanov I., Nauman M., Kumaravelu K., Lesi V., Zutshi A., Grill W.M., Pajic M. (2018). Learning-Based Control Design for Deep Brain Stimulation. Proceedings of the 2018 ACM/IEEE 9th International Conference on Cyber-Physical Systems (ICCPS).

[118] Zhu B., Farivar M., Shoaran M. (2020). ResOT: Resource-Efficient Oblique Trees for Neural Signal Classification. IEEE Trans. Biomed. Circuits Syst..

[119] De La Pava I., Mejía J., Álvarez-Meza A., Álvarez M., Orozco A., Henao O., Beltrán-Castañón C., Nyström I., Famili F. (2017). A Hierarchical K-Nearest Neighbor Approach for Volume of Tissue Activated Estimation. Progress in Pattern Recognition, Image Analysis, Computer Vision, and Applications.

[120] Bermudez C., Dawant B.M., Landman B.A., Hainline A.E., Huo Y., Rodriguez W.J., Li R., Schults R., D’Haese P.D., Konrad P.E., Angelini E.D., Landman B.A. (2019). Towards machine learning prediction of deep brain stimulation (DBS) intra-operative efficacy maps. Medical Imaging 2019: Image Processing.

[121] Park S.-C., Cha J.H., Lee S., Jang W., Lee C.S., Lee J.K. (2019). Deep Learning-Based Deep Brain Stimulation Targeting and Clinical Applications. Front. Neurosci..

[122] Ligaard J., Sannæs J., Pihlstrøm L. (2019). Deep brain stimulation and genetic variability in Parkinson’s disease: A review of the literature. Npj Park. Dis..

[123] De Oliveira L.M., Barbosa E.R., Aquino C.C., Munhoz R.P., Fasano A., Cury R.G. (2019). Deep Brain Stimulation in Patients with Mutations in Parkinson’s Disease–Related Genes: A Systematic Review. Mov. Disord. Clin. Pract..

[124] Ferrea E., Negahbani F., Cebi I., Weiss D., Gharabaghi A. (2024). Machine learning explains response variability of deep brain stimulation on Parkinson’s disease quality of life. Npj Digit. Med..

[125] Halász L., Sajonz B.E.A., Miklós G., Van Elswijk G., Hagh Gooie S., Várkuti B., Tamás G., Coenen V.A., Erōss L. (2024). Predictive modeling of sensory responses in deep brain stimulation. Front. Neurol..

